# The Role of *ARID1A* in Tumors: Tumor Initiation or Tumor Suppression?

**DOI:** 10.3389/fonc.2021.745187

**Published:** 2021-10-04

**Authors:** Shouying Xu, Chao Tang

**Affiliations:** National Clinical Research Center for Child Health of the Children’s Hospital, Zhejiang University School of Medicine, Hangzhou, China

**Keywords:** ARID1A, SWI/SNF, tumor initiation, tumor suppression, synthetic lethality

## Abstract

Genes encoding subunits of SWItch/Sucrose Non-Fermenting (SWI/SNF) chromatin remodeling complexes are collectively mutated in 20% of all human cancers, among which the *AT-rich interacting domain−containing protein 1A* (*ARID1A*, also known as *BAF250a*, *B120*, *C1orf4*, *Osa1*) that encodes protein ARID1A is the most frequently mutated, and mutations in *ARID1A* have been found in various types of cancer. ARID1A is thought to play a significant role both in tumor initiation and in tumor suppression, which is highly dependent upon context. Recent molecular mechanistic research has revealed that ARID1A participates in tumor progression through its effects on control of cell cycle, modulation of cellular functions such as EMT, and regulation of various signaling pathways. In this review, we synthesize a mechanistic understanding of the role of *ARID1A* in human tumor initiation as well as in tumor suppression and further discuss the implications of these new discoveries for potential cancer intervention. We also highlight the mechanisms by which mutations affecting the subunits in SWI/SNF complexes promote cancer.

## Introduction

Mammalian SWItch/Sucrose Non-Fermenting (SWI/SNF) complexes are ATP-dependent remodelers of chromatin structure, making chromatin more accessible for transcription factor binding (1). There are three non-redundant final form assemblies of SWI/SNF complexes: canonical BRGI/BRM-associated factor (BAF), polybromo-associated BAF (PBAF), and non-canonical BAFs (ncBAFs) (2). The BAF complex exhibits similar appearances as the SWI/SNF complex in yeast, while the PBAF complex shows more similarity with RSC-nucleosome complex (RNC) from *Saccharomyces cerevisiae* (3). SWI/SNF complexes are composed of more than 15 subunits, which are involved in transcriptional activation of genes in response to interferon β-induction (4, 5). Therein, ARID1A or AT-rich interacting domain−containing protein 1B (ARID1B) exists solely in BAF complexes, while BAF200/ARID2 and BAF180/PBRM1 are found to be exclusive in PBAF complexes. However, some core subunits are discovered in both complexes, such as BAF155/SMARCC1 and SNF5/SMARCB1 (6). Additionally, BRD9 and GLTSCR1/1L subunits are corresponding to ncBAF complexes (2).

The functional core of the SWI/SNF complex is composed of an ATPase subunit (SMARCA2 or SMARCA4) and three additional subunits (SMARCB1, SMARCC1, and SMARCC2) (7). The SWI/SNF complex remodels nucleosome structure and mobilizes nucleosomes mainly by sliding, which includes the following steps: the binding of the SWI/SNF complex to a fixed position on nucleosomal DNA and disruption of histone–DNA contacts, DNA translocation initiation through ATPase subunit, and DNA loop formation propagating around the nucleosome and generating sites more accessible to DNA binding factors (8). The SWI/SNF complex is relevant to a broad spectrum of cellular processes, such as proliferation, differentiation, damage repair, and genomic instability (9, 10). Recently, mutations of SWI/SNF complexes arise in multiple kinds of human malignancies, implying the key function of SWI/SNF complexes in tumor suppression. Initially, the SWI/SNF complex was found to be correlated with cancer in 1998 (11). Subsequently, research on cancer whole-exome high-throughput sequencing has revealed frequent mutations of genes encoding multiple subunits of the SWI/SNF complex, among which *ARID1A* is the most frequently mutated (8, 12).

**Figure 1:**
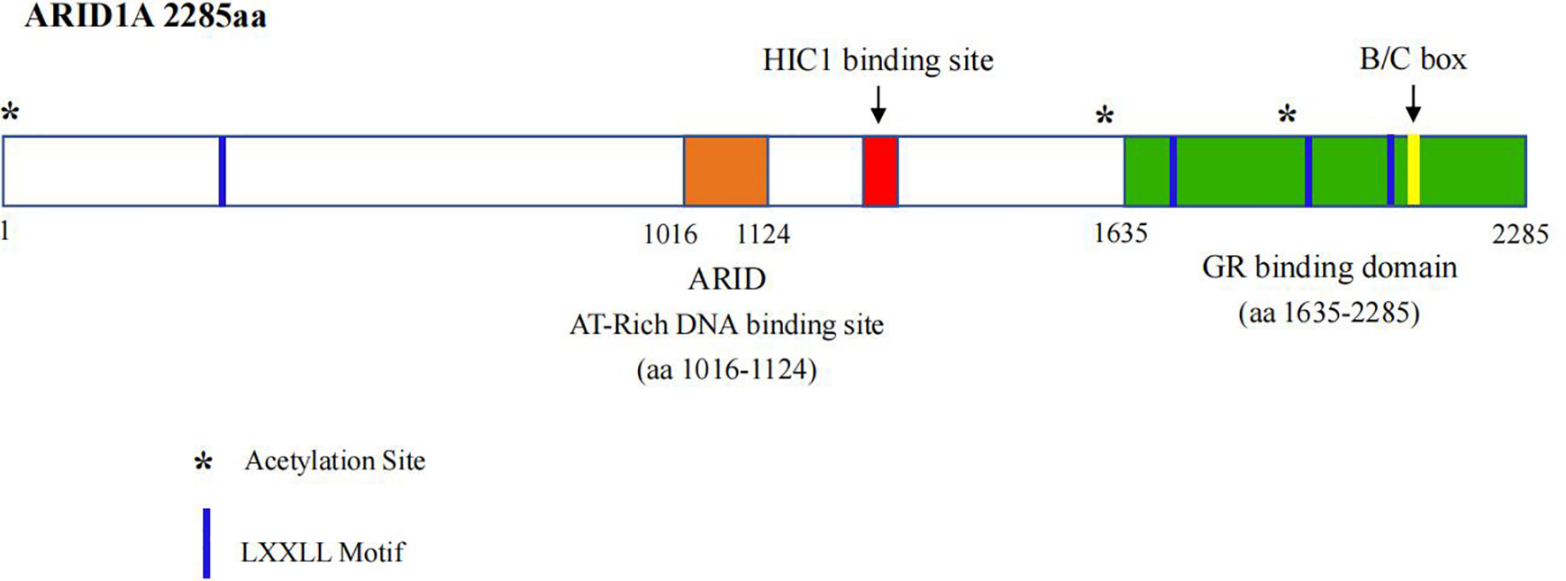
Schematic representation of ARID1A protien structure.

*ARID1A* is located in chromosome 1p35.3, whose DNA sequence contains 86,080 bp and coding mRNA contains 8,595 bp with 20 exons, and is expressed in cellular nucleus, which is evolutionally conservative and encodes a large protein ARID1A with a molecular weight of approximately 250 kDa (13). The ARID1A protein contains two conserved domains, including the N-terminal ARID domain (AT-rich interactive domain) and the C-terminal with three LXXLL motifs (**Figure 1**). The former is presumed to be crucial for enhancing BAF affinity to chromatin for certain targets *in vivo*, while the latter is identified to promote the interaction between various proteins and nuclear hormone receptors as well (14).

ARID1A plays a crucial role in mammalian development and is observed to be abundantly expressed and widely distributed in multiple stages during mouse embryonic development (15), whose loss severely impairs the pluripotency and self-renewal of embryonic stem cells and subsequently contributes to arrest in embryonic development (16). In terms of differentiation, knockout of *Arid1a* forces ES cells to differentiate into primitive endoderm, prevents their differentiation into cardiomyocytes and adipocytes (15), and gives rise to the block of differentiation of pancreatic ductal epithelial cells in mice, which is supposed to be in part through suppression of SRY-box transcription factor 9 (SOX9) expression (17). For the last few years, mutations of *ARID1A* have emerged in a broad range of cancers with high frequencies (**Table 1**) and several hot spots in *ARID1A* mutations have been identified in human cancers, including R1989, R1335, and R196 (26). Here, we focus on the role and mechanisms of ARID1A in tumors as well as on the current potential therapeutic targets.

**Table 1:** Mutation frequency of *ARID1A* gene in different human cancers.

Tumor types	*ARID1A* mutation	References
Hepatocellular carcinoma	10%–17%	(18–20)
Ovarian clear cell carcinomas	46%–57%	(21, 22)
Ovarian endometrioid carcinomas	30%	(22)
Low-grade endometrioid adenocarcinomas	47%	(23)
High-grade endometrioid adenocarcinomas	60%	(23)
Colon cancer	10%	(24)
Gastric cancer	10%	(25)

## Tumor Initiation by ARID1A

It is found that 83% of hepatocellular carcinoma (HCC) tumors possess higher ARID1A expression levels than adjacent normal tissues (16), while in endometrial cancer, the primary tumors have wild-type ARID1A, whereas the metastatic subclones carry deleterious mutations, indicating ARID1A may be required in primary cancers (27). Related studies in mouse models also imply that *Arid1a* seems to be oncogenic under highly specific conditions *in vivo*. For example, under the condition of inactivation of *Apc* and *Pten*, loss of *Arid1a* impairs the formation of ovarian cancer (28), and in an *Apc*-inactivated mouse model of colon cancer, *Arid1a* is found to be essential for tumorigenesis (29). More recently, a study by Sun et al. demonstrated that in multiple *in vivo* liver cancer models, ARID1A clearly exerts tumor-promoting functions during the early phases of transformation (30). They further established the chemically induced murine HCC models and found that *Arid1a* advances tumor initiation through cytochrome P450 (CYP450)-mediated oxidative stress (30), which provides with the possible mechanisms underlying tumor induction by ARID1A. Thus, ARID1A has a context-dependent oncogenic role in cancer.

## Tumor Suppression by ARID1A

Although ARID1A exerts cancer initiation function in certain contexts, particularly in multiple *in vivo* liver cancer models where ARID1A clearly presents tumor-promoting functions, the majority of data recently indicate that mutations in *ARID1A* usually lead to its loss of function, which makes ARID1A to be reconsidered as a tumor suppressor in a broader variety of cancers (12, 30, 31). Therefore, by discussing the involvement of ARID1A in biological processes linked to tumor suppression such as tumor formation, tumor cell proliferation, tumor cell cycle, and DNA damage repair, we herein summarize the impacts of *ARID1A* mutations in different cancers, providing a more nuanced understanding of the carcinogenic function of this frequently altered gene.

### Hepatocellular Carcinoma

Recent advancement of cancer exome sequencing has identified *ARID1A* as one of the most mutated genes in human HCC, with mutations of 10%–17% in liver cancers, and remarkably ARID1A expression levels negatively correlate with survival in HCC patients (18–20, 32). A number of studies have shown that *ARID1A* deficiency is associated with multiple mechanisms in HCC. Riou et al. found that loss of *ARID1A* in adult hepatocytes is closely related to the Wnt pathway, which can activate the transcription of erythropoietin mediated by β-catenin, thereby promoting HCC progression (33). Another study showed that deficiency of *Arid1a* affects angiopoietin‐2 (Ang2)-dependent angiogenesis, that is, *Arid1a* loss increases Ang2 expression because of augmented histone H3K27ac modification at the *Ang2* gene locus, whose upregulation confers a non-cell-autonomous growth advantage to the malignant HCC (34). Long non-coding RNAs (lncRNAs) are also reported to have critical roles during *ARID1A* loss-caused HCC development. Loss of *ARID1A* activates the transcription of *p21* (*CDKN1A*) that subsequently upregulates the expression of LncRNA MVIH (lncRNA associated with microvascular invasion in HCC), which in HCC enhances angiogenesis and serves as a potential predictor for the poor recurrence-free survival of HCC patients after hepatectomy (35).

The inhibitory effect of ARID1A on liver tumorigenesis is additionally verified by a variety of mouse models, where the hepatocyte-specific loss of *Arid1a* promotes liver tumorigenesis (30, 36). Given that *ARID1A* mutations often co-exist with *MYC* amplification in human HCC, a liver-specific tet-off mouse cancer model based on MYC overexpression and *Arid1a* knockout (*LAP-tTA;TRE-MYC;Arid1a^−/−^*) exhibits rapid preformed tumor growth, suggesting *Arid1a* deletion accelerated tumor once tumors had formed (30). In addition, the decreased expression of ARID1A in liver tumors is observed to be tightly correlated with overall metastasis such as distant metastasis, local lymph node, and poor prognosis, indicating ARID1A could be a prognostic biomarker for HCC (37, 38).

### Endometrial Cancer

Endometrial cancer has become a common gynecological malignancy globally, with approximately 280,000 cases occurring every year. According to a classification of endometrial carcinomas based upon mutations in nine genes, including *ARID1A*, *PTEN*, *PIK3CA*, *KRAS*, *P53*, and *BRAF*, the rate of *ARID1A* mutation in low−grade endometrioid adenocarcinomas is 47%, while in high−grade endometrioid adenocarcinomas, serous adenocarcinomas, and carcinosarcomas, it is 60%, 11%, and 24%, respectively (23). In addition, despite the mostly unknown genetic basis, in 14%–22% of uterine endometrial clear cell carcinoma (UCCC), a rare disease that accounts for <5% of all endometrial carcinomas, ARID1A expression is also found to be downregulated (39).

An early functional proteogenomic analysis of endometrial tumor samples with *ARID1A* mutations using reverse phase protein array demonstrates that *ARID1A* mutations often co-occur with mutations in the PI3K pathway and are related to PI3K pathway activation. In endometrial cancer cell lines, the phosphorylation of several downstream targets such as AKT and PDK1 in the PI3K pathway is markedly upregulated, which supports the view that ARID1A is a new regulator in the PI3K pathway (40). In a *Pgr^cre/+^Arid1a^f/f^;Arid1a^d/d^* mouse model, where *Arid1a* is conditionally ablated in PGR-positive cells, *Arid1a^d/d^* mice were sterile due to defective embryo implantation and decidualization with significantly increased epithelial proliferation, enhanced epithelial estrogen activity, and reduced epithelial PGR expression, which impedes maturation of the receptive uterus, indicating *Arid1a* is essential for endometrial function during early pregnancy (41). Intriguingly but noticeably, *Arid1a^d/d^* mice show aberrant active epithelial proliferation, but do not develop endometrial hyperplasia or endometrial cancer, suggesting that *Arid1a* loss alone may not be enough to cause the development of endometrial cancer. Notably, ARID1A mutation exists in the preneoplastic lesions, which occurs in the early stage of transformation from endometriosis into endometriosis-associated ovarian cancers, which may be helpful to the early diagnosis of endometrial cancer (42).

### Ovarian Carcinoma

Ovarian carcinoma is the eighth leading cause of death by cancer worldwide (43). Epithelial ovarian cancer (EOC), which accounts for over 95% of ovarian carcinoma, can be histologically classified into five major subtypes, including high-grade serous, low-grade serous, clear cell (CC), endometrioid, and mucinous ovarian cancer. Among those, the mutation rate of *ARID1A* in ovarian clear cell carcinoma (OCCC) and ovarian endometrioid carcinomas (OEC) is 46%–57% and 30%, respectively (44, 45).

In OCCC, *ARID1A*-deficient cells usually correlate with PI3K–AKT signaling tightly (46, 47). Mechanically, cancer cells lacking ARID1A expression have higher phosphorylation levels of AKT-Thr308 and AKT-Ser473, which is contrary to those in ARID1A-knockdown OCCC cell lines, indicating that phosphorylation of AKT in tumor tissues is regulated by ARID1A indirectly (46). Additionally, compared with intact mismatch repair (MMR) OCCC, *ARID1A* is more frequently lost in deficient mismatch repair (dMMR) OCCC, where the loss of MMR protein MSH2/MSH6 is the most frequent (47). Of note, similar to the case in endometrial cancer, additional molecular changes are required to collaborate with Arid1a inactivation in transforming mouse ovarian surface epithelium (MOSE), whereas the deletion of *Arid1a* alone in MOSE is not sufficient to initiate tumor development, as injection of Ad-Cre virus on ovaries in *Arid1a^f/f^* mice occurs with no gross or microscopic lesions (48).

### Gastric Cancer

Gastric cancer (GC), a heterogeneous disease characterized by epidemiologic and histopathologic differences across countries, has a higher mortality rate among cancers worldwide. GC is a multifactorial disease, and both environmental and genetic factors have a role in its etiology. In addition to the known GC driver genes including *TP53*, *PTEN*, and *CTNNB1* as well as genes reported in Catalogue of Somatic Mutations in Cancer (COSMIC) to be mutated in GC, such as *TTK* and *ACVR2A*, *ARID1A* is identified to be a new driver gene for GC development (49). In congruence with the report by The Cancer Genome Atlas (TCGA) research group showing ARID1A mutation is up to 33% in GC, results from the high-throughput sequencing further reveal that *ARID1A* is one of the most frequently mutated genes in GC with mutation prevalence ranging from 8% to 31%, and most of *ARID1A* mutation samples are accompanied by wild-type *TP53*, whose mutations and dysfunction are importantly involved in carcinogenesis (50, 51). It is noteworthy that the mutation frequency of *ARID1A* significantly varies within different molecular subtypes of gastric cancer: for microsatellite instability (MSI) type, Epstein–Barr virus (EBV) infection type, and non-EBV-infected and microsatellite stable (MSS) type, the mutation rate is 83%, 73%, and 11%, respectively (49), among which MSI and EBV types have higher PD-L1 expression, indicating ARID1A may be a potential biomarker for PD-1/PD-L1 inhibition.

More recently, mutations and protein deficiency of ARID1A have been correlated with an altered immune microenvironment in GC and responses to immune checkpoint blockade, showing *ARID1A* mutations are associated with increased immune activity (52). The diverse antitumor immune signatures are more highly enriched in *ARID1A*-mutated GC than in *ARID1A* wild-type GC, which is associated with higher tumor mutation burden and lower tumor aneuploidy level. With 68% involving CG dinucleotides, C to T transitions are the most common mutation (51%) across all gastric cancers, while *ARID1A* mutations in GC usually contain indels relating to short C or G mononucleotide repeats, implying their occurrence by mismatch defect-induced microsatellite instability (49). Of note, loss of *ARID1A* in gastric epithelial cells may be an early event in tumorigenesis, which occurs earlier than EBV infection, whereas *ARID1A* deficiency in gastric cancer cells without EBV infection or *MLH1* promotes cancer progression, suggesting that ARID1A plays different roles in gastric carcinoma (53). Given that ARID1A-mutated GC cancers expressed PD-L1 more highly than ARID1A wild-type GC cancers, which contribute to the more active immunotherapeutic responsiveness and better survival prognosis, ARID1A mutation could additionally be a useful biomarker for identifying GC cancer patients responsive to immunotherapy.

### Colon Cancer

*ARID1A* mutations are present in 10% of colorectal cancers and, similar to GC, are thought to be caused by mismatch defects. The downregulation of ARID1A is thought to induce colorectal carcinoma possibly through its regulation of proliferation and chemoresistance in colorectal cancer cells (54). In terms of mechanism, ARID1A usually targets the SWI/SNF complexes to enhancers, thereby cooperating with transcription factors to promote gene activation. *ARID1A* loss changes not only the level of histone modifications at enhancers but also their locations (29). Though H3K4me3, H3K4me1, and H3K27ac, the active *cis*-regulatory elements that are associated with histone modifications, are at SWI/SNF binding sites in *Arid1a^−/−^* cells, H3K27ac is diminished in transcription start site (TSS)-distal regions in *Arid1a^−/−^* cells at sites that lose SWI/SNF binding and is dramatically increased at the few sites where SWI/SNF binding is gained. In addition, partial *ARID1A* deficiency also affects enhancer activity, since *ARID1A^+/−^* cells show intermediate changes in H3K27ac at enhancers and in transcription of the nearest genes. On the other hand, upon loss of *ARID1A* in *Kirsten rat sarcoma viral oncogene homologue* (*KRAS*)-mutated cells, enhancers that are co-occupied by ARID1A and AP1 transcription factors become inactive, thereby leading to decreased target gene expression in colorectal cancer cells, which expands the knowledge about the context-dependent functions of ARID1A in colon cancer, particularly in *KRAS*-mutated cancer cells (55).

In *MX1-Cre;Arid1a^fl/fl^* mice, where both *Arid1a* alleles are inactivated across many tissues, nodular and polypoid tumors in the colons are obtained at multiple non-contiguous sites but never in the small intestine, whose histology is consistent with invasive colon adenocarcinoma, a malignant neoplasm derived from glandular colonic epithelium. Noticeably, in *Villin-Cre^ER-T2^;Arid1a^fl/fl^* mice, where Arid1a inactivation is restricted to intestinal epithelial cells, colon epithelium develops invasive ARID1A-deficient adenocarcinoma with prominent mucinous differentiation and presence of tumor-infiltrating lymphocytes as well as MSI, which reflects epithelial cell-intrinsic ARID1A deficiency and is associated particularly with human colorectal cancer (29, 56).

### Pancreatic Cancer

Pancreatic ductal adenocarcinoma (PDAC), generally known as pancreatic cancer (PC), ranks the fourth leading cause of cancer-related deaths in the Western world (57). Recent integrated multiplatform sequencing analyses of PDAC have revealed ARID1A mutations in 6% of the cases (58, 59). ARID1A may function as a tumor-suppressor gene in pancreatic carcinogenesis, because its expression levels are associated with tumor differentiation and tumor stage, but not to lymph node metastasis, distant metastasis, sex, or age (60). In a pancreas-specific mutant *Arid1a*-driven mouse model (*Ptf1a-Cre;Kras^G12D^;Arid1a^f/f^*), despite early development of relatively indolent cystic precursor lesions called intraductal papillary mucinous neoplasms (IPMNs), deficiency of *Arid1a* in mice pancreas develops aggressive PDAC in later ages with extensive parenchymal replacement by mucinous cysts resembling low-grade branched duct gastric type IPMN (LG-IPMN) in humans (59, 61), suggesting *Arid1a* loss accelerates PDAC formation.

### Breast Cancer

Breast cancer is a common type of cancer worldwide, which still ranks as the second most prevalent leading cause of cancer-related mortality among females (62, 63). ARID1A plays an important pathobiological role in breast cancer, and the partial loss of its expression is associated with unfavorable outcome in patients with breast cancer (64). ARID1A not only exerts an antitumor effect such as cancer cell migration and invasion on breast cancer but also enhances the sensitivity of breast cancer cells to 5-fluorouracil (5-FU) (65).

Activated estrogen receptor α+ (ER+) acts as an oncogenic signal in multiple female cancers including breast cancer by controlling tumor cell proliferation and regulating tumorigenesis through recruiting various cofactors to estrogen response elements (EREs) to mediate gene transcription (66, 67). ER binding activity is largely determined by chromatin remodeling and recruitment of transcription factors to EREs. Mechanistically, binding of estrogen to EREs leads to the dimerization of ER, which induces accessible chromatin and forms higher-order super enhancers that are pivotal to ER signaling. ER is positively related to active enhancers particularly in primary tumors, and tumors are effectively classified into molecular subtypes with their dynamics of chromatin accessibility and ER-dependent gene signature (68). Given that ARID1A binds within ER-bound enhancers and thereby regulates ER-dependent transcription, it has been suggested that the wild-type ARID1A expression is related to a better clinical outcome in ER+ breast cancer patients, where fulvestrant combined with ARID1A inhibitor could be promisingly used for ER+ breast cancer, whereas ARID1A inactivating mutations are enriched in treatment-resistant tumors and metastases (in total 12% of cases) and ARID1A inactivation is thereby considered to be a tumor-promoting event in ER+ breast cancer (69–72). Recently, ER+ breast cancer cellular plasticity, which can be defined as the ability of breast cancer cells to toggle between different phenotypes without altering genotype, is shown to be mediated by loss of *ARIDIA*-dependent SWI/SNF complex targeting genomic sites of the luminal lineage-determining transcription factors including ER, forkhead box protein A1 (FOXA1), and GATA-binding factor 3 (GATA3), and ARID1A is identified to regulate genome-wide ER–FOXA1 chromatin interactions and ER-dependent transcription as well, also indicating the role of ARID1A in maintaining luminal cell identity and endocrine therapeutic response in ER+ breast cancer (73).

### Choriocarcinoma

Choriocarcinoma is a rare but highly malignant cancer characterized by highly pro-angiogenic and metastatic malignant epithelial tumors, which can be divided into gestational and non-gestational types based on differences in the onset of symptoms (74). Our recent study reported that ARID1A is expressed in different choriocarcinoma cell lines including JEG-3, JAR, and BeWo (75). We found that overexpression of ARID1A suppresses choriocarcinoma cell migration and invasion, whereas inhibition of ARID1A promotes migration and invasion in choriocarcinoma cells, suggesting the tumor-suppressor function of ARID1A in choriocarcinoma progression.

### Summary

In most cases, ARID1A has emerged as a cancer suppressor in a broad array of cancers, such as HCC and ovarian carcinoma. *ARID1A* loss in cancer cells leads to increased cell proliferation, migration, and invasion, as well as reduced cell apoptosis and chemosensitivity. Consistent with the high mutation frequencies of *ARID1A* in many cancer types, ARID1A has been identified to exert a variety of tumor-suppressive functions during carcinogenesis. In particular, in histological or molecular subtypes of cancer, *ARID1A* mutations usually occur with high specificity. Altogether, we demonstrated that ARID1A biochemically, functionally, and clinically participates in tumor progression and potential treatment strategies across many types of human cancer. Up to now, several studies have revealed the synthetic lethal targets with ARID1A deficiency as a potential strategy for cancer treatment.

## Underlying Mechanisms of Tumor Suppression by ARID1A

*ARID1A* mutations are specific in cancers of different tissue subtypes, which is due to the variations in mutation-corresponding mechanisms (24). Below, we summarize the common mutations in different genes cooperating with *ARID1A* in related signaling pathways (**Table 2**).

**Table 2:** Signaling pathways related to the roles of ARID1A in tumor initiation and tumor suppression.

Roles of ARID1A	Related pathways	Genetic background	Citations
Tumor initiation	PI3K/AKT pathway, Wnt pathway	Conditional inactivation of Arid1a concurrently with Apc and Pten in ovarian cancer mouse model	(28)
	Wnt pathway	ARID1A is necessary to promote tumorigenesis driven by APC inactivation	(29)
Tumor suppression	PI3K/AKT pathway	ARID1A inhibits PIK3CA and PDK1 transcription by directly binding to the promoters in GC	(50)
		ARID1A depletion improves radioresistance of pancreatic cancer *via* activation of the PI3K/AKT pathway	(76)
		Conditional knockout of Arid1a and Pten in mouse model drives tumorigenesis of MOSE	(77)
	YAP/TAZ pathway	ARID1A-SWI/SNF complex acts as an inhibitor of YAP and TAZ2	(78)
	Wnt pathway	Loss of ARID1A in adult hepatocytes activates the transcription of erythropoietin mediated by β-catenin	(33)
	IFN pathway	The interaction between ARID1A and EZH2 antagonizes EZH2-mediated IFN responsiveness	(79)
	IL-6/STAT3 pathway	Hepatocyte-specific Arid1a deletion could lead to mouse steatohepatitis and HCC development	(36)

### PI3K/AKT Pathway

The PI3K/AKT pathway is abnormally activated in a large fraction of cancers and controls the majority of cellular processes, such as cell growth, cell proliferation, metabolism, and genome stability. Approximately 80% uterine endometrioid adenocarcinomas present abnormality of the PI3K/AKT pathway (80). In many cancers, mutation of *ARID1A* has been found to co-occur with mutations in the PI3K/AKT pathway (40, 50, 81, 82), among which the coexistence of *ARID1A* deficiency and phosphatidylinositol 3-hydroxy kinase (PIK3CA) mutation is a more typical phenomena. For example, in gastric adenocarcinoma, *ARID1A* mutations are correlated with simultaneous *PIK3CA* mutations in 8% of tumors (50). In *ARID1A*-mutated OCCC, PIK3IP1 is recognized as a direct target of ARID1A and Zust homologous enhancer 2 (EZH2), which is upregulated by EZH2 inhibition that suppresses PI3K–AKT signaling (83).

### YAP/TAZ Pathway

Yes-Associated Protein (YAP) and Transcriptional Co-activator with PDZ-binding Motif (TAZ), two key effectors in Hippo signaling, have both emerged as important drivers of cancer progression and metastasis. ARID1A binds to YAP/TAZ and prevents their binding to the TEA-Domain transcription factor (TEAD), leading to failure in induction of transcription of downstream target genes by YAP/TAZ and subsequent inhibition of tumorigenesis mediated by YAP/TAZ, and on the contrary, YAP/TAZ are necessary to induce the effects of the inactivation of the ARID1A-containing SWI/SNF complex (78). In the mouse model, knockout of *ARID1A* and *neurofibromin 2 (NF2)* genes in the liver results in upregulation of YAP and significant enlargement of liver volume as well as tumorigenesis, also verifying the intranuclear inhibitory effect of ARID1A on YAP/TAZ (78).

### ARID1A and Cell Cycle Arrest

ARID1A-containing SWI/SNF complexes have been discovered to regulate the expression of cell cycle-specific genes and be critical for cell cycle arrest (24). ARID1A is accumulated in the G0 phase and is downregulated at various stages of the cell cycle. Due to the influence of cycle changes, ARID1A is thought to be almost completely absent in cells that divide vigorously (84, 85). When DNA is damaged, ARID1A initiates and maintains cell cycle arrest, thereby preventing the damaged DNA from being passed on to the next cell cycle (86). ARID1A was reported to directly or indirectly regulate the expression of the *p21/WAF1* gene, which promotes cell cycle arrest in G1 phase by binding to the cyclin–CDK2/CDK4 complex. On the other hand, knockdown of ARID1A upregulates the expression of cycle-related proteins cyclinD1 and Bcl-2 and inhibits cell apoptosis in non-small cell lung cancer (NSCLC) (87).

Modulating p53 expression resulted in cell cycle arrest at low p53 levels and apoptosis at higher levels. In the same tumor, simultaneous mutation of *ARID1A* and *TP53* is rarely observed (88). The correlationship between *ARID1A* and *TP53* mutations has been found in many kinds of cancers, such as gastric carcinoma (49, 50), uterine endometrioid carcinoma (81, 89), and esophageal adenocarcinoma (90). ARID1A has been demonstrated to interact with p53 *via* its C-terminal region and stimulate transcriptional activity (14, 91). In an ovarian cancer cell line model, ARID1A interacts with p53 directly to regulate the transcription of such p53 target genes as *p21*, leading to the induction of p21 and subsequent cell cycle arrest (92). These lines of evidence suggest that ARID1A and p53 suppress cancer downstream genes *via* transcriptional activation to cooperatively inhibit carcinogenesis.

### ARID1A and Hormone Signal Regulation

ARID1A stimulates transcriptional activation *via* steroid hormone receptors, such as androgen and estrogen receptors. A recent study demonstrates that ARID1A plays a role in glucocorticoid-induced transcriptional co-activation in ovarian carcinomas (93). In breast cancer cells with *ARID1A* deficiency, ARID1A re-expression is believed to enhance the transcriptional activity through glucocorticoid, estrogen, and estrogen receptors (12). So far, it is not clear whether the effect of ARID1A on hormone signaling is related to tumor suppression, although *ARID1A* mutations are often present in cancers that occur in hormone-responsive tissues, such as the breast and the ovary (12).

### ARID1A and Epithelial–Mesenchymal Transition

Epithelial–mesenchymal transition (EMT) refers to the biological process in which cells lose epithelial properties and acquire mesenchymal phenotypes (94). The main characteristics of EMT include the decrease in the expression of cell adhesion molecules (such as E-cadherin) and the formation of vimentin-based cytoskeleton (95). ARID1A affects the process of EMT in multiple cancers, such as gastric cancer, neuroblastoma, and liver cancer (37, 96–98). In gastric cancer cell lines, the silence of ARID1A increases the expression of vimentin and N-cadherin and promotes local lymph node metastasis and distant metastasis (96, 97). Meanwhile, knockdown of ARID1A in neuroblastoma SK-N-SH cells is found to raise the expression of matrix metalloproteinase-2 (MMP-2) and matrix metalloproteinase-9 (MMP-9) and lessens the expression of E-cadherin, which ultimately leads to the enhancement of neuroblastoma invasion and migration (98), and the upregulated levels of MMP-9 upon ARID1A inhibition is recently shown to be due to the strengthened MMP-9 protein stability in JEG-3 choriocarcinoma cell line (75). Additionally, in mouse endometrial epithelial cells, ARID1A can bind to the promoter region of EMT-related genes to inhibit the occurrence of EMT (89). However, when ARID1A is mutated, this inhibition is lifted and the expression of EMT-related genes is increased (89).

### ARID1A and MMR Deficiency

MMR is a form of DNA repair mechanism, which mainly corrects base mismatches to prevent genetic mutation and maintain genomic stability (99). The loss of MMR protein can lead to the accumulation of DNA replication errors, especially in the region of the short tandem repeat genome, which is called MSI (100). Loss of *ARID1A* is closely associated with *MMR* deficiency and MSI in gastric cancer, colorectal cancer, and uterine endometrioid carcinoma (49, 53, 88, 101). Tumors with abnormal expression of ARID1A exhibit functional defects of the *MMR* genes closely related to clinical research such as *MLH1*, *MSH2*, *MSH6*, and *PMS2*, and tumor phenotypes with high microsatellite instability (MSI-H) are mainly characterized by changes in these genes. ARID1A inactivation is related to lymphatic infiltration, lymph node metastasis, poor prognosis, and MMR defects in gastric adenocarcinoma (102). Intriguingly, in endometrial cancer, *ARID1A* is a pathogenic gene of MSI rather than a target gene due to its function in the epigenetic silence of the *MLH1* gene.

### Summary

The tumor suppression mechanisms of ARID1A interplay with other oncogenic pathways and affect the proliferation and growth of cancer cells. By far, *ARID1A* mutation has been found in cooperation with several processes associated with cancer development and progression, including the PI3K/AKT pathway, cell cycle arrest, hormone signaling regulation, and MMR deficiency. Based on *ARID1A* mutation status, several synthetic lethality targets have been discovered, with candidates in clinical trials developing now. Further studies are required to explore the molecular mechanisms of ARID1A at different stages of carcinogenesis in different cancers, which may help reveal the tumor-initiation or tumor-suppression functions of ARIDIA.

## Potential ARID1A Synthetic Lethal Targets

**Figure 2:**
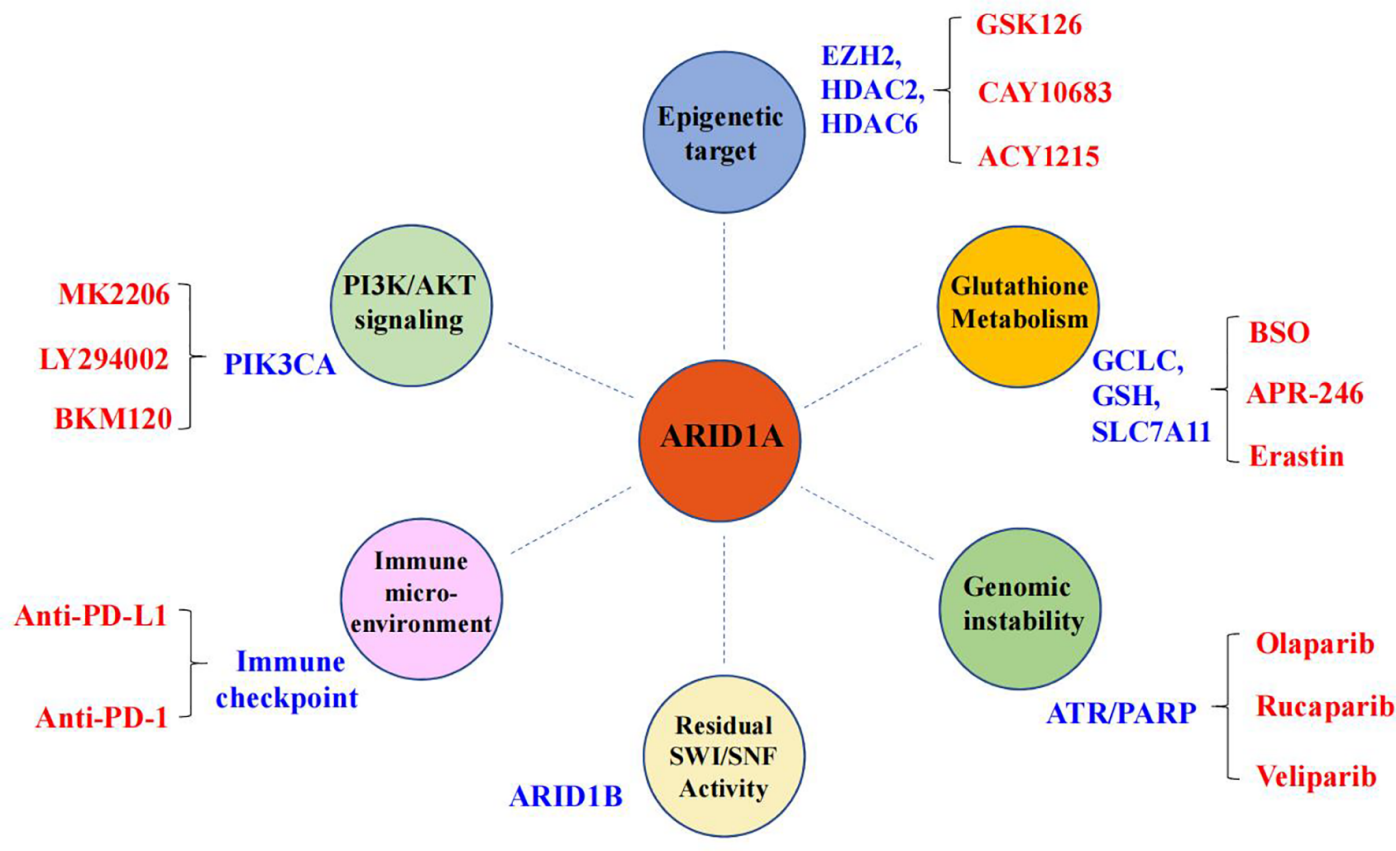
Synthetic lethal targets of ARID1A deficiency. The drug targets are listed in blue, and the specific names of drugs are labeled in red.

It is impossible for tumor-suppressor genes to directly restore their specific functions in cancer patients, but deletion of tumor-suppressor genes can become a specific site for targeted therapy, such as the synthetic lethal (SL) strategy. Conceptually, SL can be described as the scenario where loss of gene *A* or loss of gene *B* is compatible with cellular viability, while concurrent loss of both gene *A* and gene *B* is lethal to the cell, provoking cell death (103). In recent years, SL strategies have been introduced to treat tumors with loss of tumor-suppressor genes. So far, based on the mutational status of *ARID1A*, multiple therapeutic targets have been extensively studied (**Figure 2**), such as poly (ADP-ribose) polymerase (PARP), EZH2, PIK3CA, the glutathione metabolic pathway, and histone deacetylase 6 (HDAC6) (83, 85, 104–109). Currently, clinically applicable drugs targeting EZH2 or inhibiting PI3K/AKT signaling have been developed (104). Here, we will highlight the progress made in identifying therapeutic targets for ARID1A mutant cancers.

### Poly (ADP-Ribose) Polymerase Inhibitors

PARP have been proved to be a new target in cancer therapy in nearly a decade, and PARP inhibitors stand for a reinvented example of precision medicine as the first drugs targeting DNA damage response to have triumphantly entered the clinic (110, 111). PARP inhibitors are generally used to treat homologous recombination (HR)-deficient cancers, including dysfunction of *BRCA1/2* or other genes in the HR pathway.

By now, four PARP inhibitors have been approved by the U.S. Food and Drug Administration (FDA) and by the European Medicines Agency (EMA), namely, olaparib, rucaparib, niraparib, and talazoparib. Among those, olaparib was firstly approved as the maintenance therapy in 2014, particularly for platinum-sensitive advanced ovarian cancer with germline mutations in *BRCA1/2* genes that are involved in the HR pathway of DNA double-strand break (DSB) repair. Then, rucaparib was approved in 2016 for the treatment of advanced ovarian cancer with both germline and somatic mutations in *BRCA1/2* genes. Later in 2017 and 2018, olaparib, rucaparib, and niraparib were approved for the maintenance therapy for recurrent cancers irrespective of the *BRCA* gene status, such as epithelial ovarian cancer, fallopian tube cancer, and primary peritoneal cancer. Olaparib and talazoparib were approved recently in 2018 for human epidermal growth factor receptor type 2 (HER2)-negative locally advanced or metastatic breast cancer with germline mutations in *BRCA1/2* genes. It is noteworthy that multiple clinical trials have been carried out since 2019, demonstrating the efficacy of PARP inhibitors not only limited to *BRCA* gene-mutated ovarian cancer and breast cancer but also in prostate cancer, pancreatic cancer, and small cell lung carcinoma (SCLC), irrespective of the *BRCA* gene status (110, 112–118).

PARPs synthesize poly (ADP-ribose) (PAR) from NAD and release its reaction product, nicotinamide (119). As the major producer of cellular PAR, PARP1 is activated by its binding of DNA lesions. Catalytic activation of PARP1 is a multistep process, which includes binding to DNA through N-terminal zinc fingers (ZnF), unfolding of the helical domain (HD), binding of NAD to the catalytic pocket, and PAR catalysis (120). Intriguingly, the first PARP1 inhibitor was nicotinamide itself, which was followed by 3-aminobenzamide (3-AB) (121). Thereafter, all subsequently developed PARP1 inhibitors contain nicotinamide/benzamide pharmacophores and compete with NAD for the catalytic pocket of PARPs. Mechanismly, PARP1 inhibitors function by docking into the catalytic site, where they form hydrogen bonds with Gly, Ser, and Glu and interact with the two hydrophobic stacking Tyr residues within the nicotinamide-binding pocket (122). Considering the high degree of conservation of the catalytic pocket among different PARPs, additional interactions are expected to be required for further selective inhibition by the PARP1 inhibitors (123). Later by screening, different scaffolds were identified fortunately, including the new-generation PARP1 inhibitors, among which phthalazinone and tetrahydropyridophthalazinone served as a scaffold for olaparib and talazoparib, benzimidazole and indazole carboxamide for veliparib and niraparib, and tricyclicindole lactam for rucaparib, respectively (124, 125). Data show that all the clinically relevant PARP1/2 inhibitors possess high catalytic activity with IC50 in the low nanomolar range and inhibit PARP1 and PARP2 with similar efficiency, among which veliparib is the most selective PARP1/2 inhibitor, followed by niraparib, while rucaparib is the least selective clinical PARP1 inhibitor that inhibits different PARPs (PARP1, PARP2, PARP5A, and PARP5B) as well as mono (ADP-ribosyl) transferases including PARP3, PARP4, PARP10, PARP15, and PARP16 (126–129). In addition, despite of their lower efficiency, some PARP inhibitors, such as rucaparib and niraparib, also present inhibitory functions against non-PARP targets (130). Although the four clinically relevant PARP inhibitors, namely, olaparib, rucaparib, niraparib, and talazoparib, exhibit similar catalytic inhibitory effects, their potency varies considerably in trapping PARP–DNA complexes, and veliparib is one of the weakest PARP1/2 inhibitors with low PARP trapping efficiency (131, 132).

PARP1 interacts with DNA replication machinery and is active during S phase and in response to replication stress. ATR, the DNA damage checkpoint kinase, suppresses silent origin firing and activates the checkpoint kinase CHK1 to induce cell cycle arrest (133). Recent studies revealed that *ARID1A*-deficient tumors are sensitive to PARP inhibitors and ATR inhibitors, and the combination of PARP inhibitors and ionizing radiation in the treatment of tumor cells with *ARID1A* deficiency has a selective and high efficiency (86, 134). Mechanically, ARID1A interacts with ATR and promotes the effective processing of DNA DSBs to single-strand ends, sustaining DNA damage signal transduction (86). In addition, *ARID1A* loss sensitizes cancer cells to PARP inhibitors potentially *via* inhibition of ATR function in DSB repair, suggesting a new method of targeting tumor cell synthesis to lethality.

Nevertheless, PARP inhibitors cause cytotoxicity, which was found to correlate with the strength of PARP–DNA entrapment rather than a reduction in PARP1 catalytic activity (135). The physical obstruction induced by PARP–DNA entrapment exacerbates replication problems caused by loss of PARP activity and induces mitotic phenotypes of premature loss of cohesion or anaphase DNA bridges. Moreover, resistance to chemotherapy is a frequent problem in clinical practice that also affects PARP inhibitors, and the most common avenue of PARP inhibitor resistance is restoration of the HR pathway (136, 137). For example, PARP inhibitor resistance was identified in Capan-1 cells derived from a pancreatic epithelial tumor (138). Thus, further investigation of understanding on a molecular and cellular level how PARP maintains replication fork integrity and how replication stress and genomic instability resulting from its inhibition instigate mitotic defects and cell death by replication and mitotic catastrophe would advance new biomarkers of PARP inhibitor sensitivity and would guide new combination strategies.

### PI3K/AKT Pathway Inhibitors

Based on the primary structure and lipid substrate specificity, PI3Ks are divided into four classes, namely, class I, class II, class III, and class IV. Among those, class II PI3Ks produce phosphatidylinositol 3-phosphate [PI(3)P] from PI and phosphatidylinositol 3,4-bisphosphate [PI(3,4)P_2_] from phosphatidylinositol 4-bisphosphate [PI(4)P], while class III PI3Ks catalyze the production of [PI(3)P] from PI (139–141) and class IV PI3Ks are a group of distantly related Ser/Thr protein kinases, including ataxia telangiectasia mutated (ATM), ataxia telangiectasia and Rad3-related (ATR), DNA-dependent protein kinase (DNA-PK), and mammalian target of rapamycin (mTOR) (142). Only class I PI3Ks, heterodimers that consist of a catalytic subunit and a regulatory subunit, are involved in the production of phosphatidylinositol (3,4,5)-trisphosphate (PIP_3_) and activation of AKT and can be further divided into class IA that consists of a p110 catalytic subunit and a p85 regulatory subunit and class IB that is composed of a p110γ catalytic subunit and a p101 regulatory subunit (143–145). AKT, also named protein kinase B (PKB), is a serine/threonine kinase and a primary mediator in PI3K signaling cascade (146). To date, three highly conserved AKT isoforms are identified, namely, AKT1 (AKT), AKT2, and AKT3, among which AKT is recognized as a cardinal node in diverse signaling pathways with a wide range of downstream substrates, such as forkhead family of transcription factors (FOXO), IκKα, GSK3β, MDM2, procaspase-9, Bim, and Bad (147). Upon activation, AKT not only activates mTOR signaling by direct phosphorylation at Ser2448 of mTOR but also catalyzes phosphorylation at Thr1462 of TSC2 that inhibits TSC1/TSC2 complexes, a master negative regulator of mTOR (148, 149). Therefore, AKT functions in the activation of mTOR signaling through both direct phosphorylation of mTOR protein and inhibition of the TSC1/TSC2 complex.

Over the past several decades, strenuous efforts have been made in the development of new targeted therapy using small molecule inhibitors against the PI3K/AKT pathway, which specifically targets toward PI3K, AKT, or mTOR. Among those, over 40 compounds have been tested by clinical application in a series of cancers, but further clinical development of many small molecule chemicals was terminated before late-phase randomized trials due to the limited antitumor activity and/or prohibitive host toxicities. By now, only two PI3K inhibitors (idelalisib targeting PI3Kδ and copanlisib targeting Pan-PI3K) and two mTOR inhibitors (temsirolimus targeting mTORC1 and everolimus targeting mTORC1) have been approved by the FDA for the clinical treatment of cancers (150), whereas AKT inhibitors are still in the early phase of clinical tests, and agents that target the AKT E17K mutant appear to be encouraging (151).

As a selective inhibitor of the PI3K catalytic subunit δ, idelalisib was the first PI3K inhibitor approved for the treatment of chronic lymphocytic leukemia (CLL) in combination with rituximab. Compared with rituximab alone, this therapy obviously improves overall response rate (ORR), progression-free survival (PFS), and overall survival (OS) of CLL patients; however, grade 3 or 4 adverse events occurred in 56% of patients in the idelalisib arm, including severe diarrhea, rash, a high risk of serious immune-mediated hepatotoxicity, pneumonitis, and infection (152). Copanlisib is a pan PI3K inhibitor that received accelerated approval from the FDA for the treatment of refractory follicular lymphoma in September 2017, and hyperglycemia and nausea are the major side effects of copanlisib (153).

PI3K/AKT pathway inhibitors, such as the AKT inhibitor MK2206 and PI3K inhibitors LY294002 and BKM120, have been demonstrated to be effective to inhibit proliferation and growth in *ARID1A*-loss cancer cells (154, 155). Meanwhile, in pancreatic cancer, the application of PI3K/AKT pathway inhibitors enhances cell apoptosis, which in turn leads to a significant increase in radiosensitivity of *ARID1A*-deficient pancreatic cancer cells (76), and the sensitivity of *ARID1A*-depleted cancer cells to treatment with inhibitors of the PI3K/AKT pathway is increased by the loss of ARID1A expression (106). Mechanically, ARID1A deficiency increases the phosphorylation of AKT at Ser-473 (pAKT-Ser^473^), as well as the downstream phosphorylation of p70S6kinase (pS6K) (106). Thus, small molecular inhibitors against the PI3K/AKT signaling pathway, such as idelalisib and copanlisib, may be used as potential targeted therapeutic drugs for tumors where *ARID1A* is deficient.

To date, only a few agents are approved by the FDA for clinical use, but overall response rate is low, resistance emerges quickly, and safety concerns arise due to on-target and off-target toxicity that often causes limitation of exposure doses. Considering the potential strategies that may be applied to improve the PI3K/AKT-targeted cancer therapy, development of predictive markers and personalization of each treatment may improve the objective response rate of PI3K/AKT inhibitors and patient survival.

### Enhancer of Zeste Homolog 2

As a histone methyltransferase that functions as the catalytic subunit of the polycomb repressive complex 2 (PRC2), EZH2 inhibits the functions of Th1 chemokine C-X-C motif chemokine ligand 9 (CXCL9) and C-X-C motif chemokine ligand 10 (CXCL10) and exerts a carcinogenic effect in a variety of tumors, including breast cancer, ovarian cancer, prostate cancer, non-Hodgkin lymphoma (NHL), and T-cell ALL (156–159). Several small molecules that suppress the enzymatic activity of EZH2 have been recently developed. While most compounds are still in preclinical development, three agents (tazemetostat, GSK2816126, and CPI-1205) have entered phase I/II clinical trials.

A high-throughput screening of a chemical diversity library against PRC2 performed in 2012 identified a molecule EPZ 005687, and later appeared tazemetostat, an orally bioavailable small molecule inhibitor of EZH2 that was optimized from EPZ 005687 (160, 161). Tazemetostat inactivates EZH2 through competitive inhibition with the cofactor S-adenosyl-L-methionine (SAM) that is required for the function of EZH2, and tazemetostat inhibits both wild-type and mutant forms of EZH2 with an IC50 ranging from 2 to 38 nM (157). In addition, tazemetostat has been tested in solid tumors with notable preclinical activity in pediatric malignant rhabdoid tumors, which harbor inactivating biallelic mutations in the SWI/SNF subunit SMARCB1 as well as SMARCB1-deificent synovial sarcoma (157, 162). Similar to tazemetostat, GSK2816126, a small molecular inhibitor against EZH2 that was generated through chemical optimization of a compound identified from a high-throughput biochemical screening of compounds targeting EZH2, inhibits both WT and mutant EZH2 through competitive inhibition with SAM (163, 164). CPI-1205, an orally bioavailable, indole-based, small molecule inhibitor of EZH2, is an N-trifluoroethylpiperidine analog of the chemical probe CPI-169 that binds to the EZH2 catalytic pocket and partially overlaps with the SAM binding site (165, 166).

The EZH2 inhibitor selectively suppresses the growth of ARID1A-mutated cells, which is neither due to changes in EZH2 expression nor due to inability of the EZH2 inhibitor to suppress the enzymatic activity of EZH2 in ARID1A wild-type cells (161, 163, 167). Similarly, in diffuse large B-cell lymphoma (DLBCL), the response to EZH2 inhibitors often correlates with gain-of-function mutations in EZH2. A recent study shows that *ARID1B* is a specific vulnerability in ARID1A mutant cancers, further highlighting the potential of synthetic lethal strategies for *ARID1A* mutation in cancer (86). However, *ARID1A* mutation impairs the expression of *IFN* and defines the chromatin accessibility to IFN-responsive genes (79). Mechanistically, ARID1A interacts with EZH2 *via* its C-terminal region and antagonizes EZH2-mediated IFN responsiveness, and the DUF3518 domain of ARID1A is essential for tumor cell IFN-γ response (79). Intriguingly, the R1989* nonsense mutation in the DUF3518 domain in ARID1A has been identified in various human cancers (26). Moreover, in *ARID1A*-deficient OCCC, EZH2 methyltransferase inhibitors have been reported to act in a synthetically lethal manner, causing *ARID1A*-mutant ovarian tumors to be regressed in tumor-bearing mice (83). With an increasing understanding of the role that EZH2 plays in oncogenesis and encouraging data from clinical trials, additional EZH2 inhibitors continue to be synthesized and evaluated in the preclinical setting, and the mutation status of *ARID1A* may become a biological marker with clinical value.

### Histone Deacetylase 6

Histone deacetylases (HDACs) regulate extensive cellular processes by modulating protein structure and regulating protein function correspondingly *via* lysine deacetylation (168, 169). As a post-translational modification that is theorized to play key roles in cellular signaling and homeostasis similar to protein phosphorylation, lysine deacetylation is a dynamic and reversible process mediated by acetyl transferases and HDACs (170, 171). The initial classification of human HDACs is based on sequence homology and phylogenic relationships to yeast homologs. To date, four distinct classes of HDACs have been clarified, namely, class I HDAC (1, 2, 3, and 8), class IIa HDAC (4, 5, 7, and 9), class IIb HDAC (6 and 10), and class IV HDAC (11) that represents the Zn^2+^-dependent deacetylases (172). The class III sirtuins are mechanistically diverse NAD^+^-dependent deacetylases (173).

As an epigenetic protein that deacetylates diverse substrates, HDAC6 is important for regulating protein trafficking, cell shape, and migration. Tubacin, the HDAC6 selective inhibitor, has been shown to selectively induce apoptotic cell death in multiple myeloma and acute lymphoblastic leukemia (AML) cells with a lesser effect on normal peripheral blood mononuclear cell (PBMC) (174, 175). Similarly, NQN-1, a compound derived from the screen targeting HDAC6, displays strong potency and selectivity at inducing AML cell death and HDAC6 inhibition, suggesting HDAC6 inhibition in the treatment of AML (169).

Previous studies presented that inhibition of HDAC6 markedly suppresses the growth of *ARID1A*-mutated tumors and has immunomodulatory effects on tumor immune microenvironment (176–178). A more recent study reveals that in ARID1A deficiency ovarian cancer, HDAC6 inhibition synergizes with anti-PD-L1 immune checkpoint blockade, and ARID1A directly repressed the transcription of the *CD274* (encoding PD-L1) gene (108). Utilizing Arid1a-mutated OCCC mice treated with the HDAC6 inhibitor and anti-PD-L1 immune checkpoint blockade, they further found that tumor burden reduced and mice survival improved because of the activation and increased presence of interferon-gamma-positive CD8 T cells. Collectively, these findings indicate that HDAC6 inhibition and immune checkpoint blockade may be used in combinational clinical application for ARID1A-mutant tumors.

### Glutamate-Cysteine Ligase Synthetase Catalytic Subunit

GCLC is a rate-limiting enzyme for antioxidant glutathione (GSH) synthesis and shows all the catalytic activities of the separated enzyme and the feedback inhibition of GSH (179). In *ARID1A*-deficient cancer cells, GSH level decreases due to the impairment of solute carrier family 7 member 11 (*SLC7A11*), the gene encoding cystine transporter that is required for incorporation of cystine and supplies cells with cysteine, a key source of GSH. Inhibition of GCLC leads to apoptotic cell death in *ARID1A*-deficient cancer cells (109), and the vulnerability of *ARID1A*-deficient gastric cancer cells to GSH inhibition is caused by decreased GSH synthesis due to diminished SLC7A11 expression (180). Similarly, *ARID1A*-deficient OCCC cells are sensitive to GSH inhibitors such as APR-246, as well as to BSO (buthionine sulfoximine), which inhibits GCLC, where high levels of ROS (reactive oxygen species) are accumulated (181). Thus, GSH inhibition, such as synthetic lethal targeting of GCLC, is a prospective therapeutic strategy for *ARID1A*-deficient tumors.

### Summary

Since the critical tumor-suppression effect of ARID1A has been unraveled, several studies have attempted to identify potential strategies for ARID1A-mutated cancer treatment based on synthetic lethal targets. By far, several therapeutic targets with the potential of specifically targeting ARID1A-mutated cancers have been revealed. Therein, some drugs like EZH2 inhibitors are now clinically applicable. Further research is needed to investigate the potential side effects and pharmacodynamics of these proposed combinational strategies. Thus, ARID1A mutation has potential as a biomarker for precision medicine of related cancers.

## Other SWI/SNF Members and Cancer

SWI/SNF complexes are critical in modulating gene transcription through remodeling nucleosomes. In addition to *ARID1A*, the mutations in other SWI/SNF members such as *ARID1B*, *SMARCA4*, and *SMARCB1* are also identified in multiple kinds of human malignancies (**Table 3**), implying the vital role of other subunits in SWI/SNF complexes in tumorigenesis.

**Table 3:** Other SWI/SNF members involved in tumorigenesis and progression.

SWI/SNF subunits	Genes	Molecular weight (kDa)	Domains	Associated cancers (mutation frequency)
BRG1	*SMARCA4*	184.5	Bromo, ATPase, HAS, QLQ	Ovarian cancer (>10%), small cell cancer of the ovary (>90%), non-small-cell lung cancer (35%), medulloblastoma (5%–10%)
BAF250b	*ARID1B*	236	ARID	Clear cell ovarian (>10%), childhood neuroblastoma (7%), liver cancer (5%–10%)
BAF47	*SMARCB1*	44	SNF5	Rhabdoid tumor (>98%), familial schwannomatosis (30%–45%), epithelioid sarcomas (>55%), small-cell hepatoblastomas (36%)
BAF180	*PBRM1*	193	Bromo, HMG	Epithelioid sarcoma (83%), renal cancer (~40%)
BRD7	*BRD7*	74	Bromo	Breast cancer
BAF200	*ARID2*	197	ARID, zinc finger	Liver cancer (5%–14%), melanoma (5%–15%)
BRM	*SMARCA2*	181	Bromo, ATPase	Rhabdoid tumor (60%), gastric (15%), breast (15%), and lung (4.8%–10%) cancers
BAF155	*SMARCC1*	123	Chromo, SANT, BRCT	Prostate cancer (~30%)

The mutation frequencies of different SWI/SNF subunits in associated cancers are summarized here using data from various publications (182–186).

### ARID1B

ARID1B, a homolog of ARID1A, has a high degree of sequence similarity with ARID1A (84). Compared with ARID1A, ARID1B possesses a lower mutation rate. The BAF complex plays an important role in cell cycle regulation, and the dynamics of ARID1A and ARID1B are different over the cell cycle: ARID1B is accumulated in the entire cell cycle, even during mitosis, whereas ARID1A is synthesized in G0 phase but decreased throughout the cell cycle phase (85, 187). In terms of cell cycle arrest, ARID1B has the opposite functions to ARID1A: ARID1A is required for cell cycle arrest upon serum deprivation, whereas ARID1B is needed for serum-stimulated cells to re-enter into the cell cycle (24). Intriguingly, these two homologs are mutually exclusive since SWI/SNF complexes only contain either one of them (8). *ARID1B* is a preferred gene for the survival of *ARID1A*-mutant tumor cell lines, while loss of *ARID1B* in the background of *ARID1A* mutation destabilizes the SWI/SNF complex and impairs cell proliferation (82).

### Brahma-Related Gene 1

*Brahma-related gene 1* (BRG1, also known as *SMARCA4* or *BAF190A*) encodes a catalytic ATPase subunit of the SWI/SNF chromatin remodeling complex, which can shift the position of nucleosomes by using the energy derived from ATP hydrolysis. In keeping with a vital role for the SWI/SNF chromatin remodeling complex in tumorigenesis, *BRG1* is found to be frequently mutated or deleted in various types of human cancers such as non-small-cell lung cancer and ovarian small cell carcinoma (188, 189). Particularly, in these cancer types, mutations in *BRG1* display loss of function phenotypes and *BRG1* appears to function as a tumor suppressor in these tissue settings accordingly. In hypercalcemic type of small cell carcinoma of the ovary (SCCOHT), the mutation rate of *BRG1* is over 90%, indicating *BRG1* is a critical tumor suppressor. Loss of *BRG1* is exceedingly specific and sensitive for SCCOHT, accompanied by loss of *SMARCA2*, which is due to its absence of mRNA expression rather than genetic mutation (190), while restoration of either BRG1 or SMARCA2 expression could inhibit the growth of SCCOHT cell lines.

Similar to *ARID1A*, the regulation of *BRG1* in tumorigenesis of different cancers is rather complicated, which is considered to be tissue type and cellular context dependent (191). In pancreatic cancer setting, BRG1 exhibits both tumor-suppressive and tumor-promoted roles at distinct stages of pancreatic cancer formation, presenting a cellular context-dependent manner. Of note, BRG1 is significantly overexpressed in some human cancer types including breast cancer, medullablastoma, and acute leukemia, where BRG1 is found to be essential for promoting tumor cell proliferation and clinically high expression of BRG1 is usually correlated with poor outcome (192–194). For instance, BRG1 modulates a different set of gene expression from those in non-small-cell lung cancers (195), while in the gastric cancer setting, Sentani et al. observed no genetic mutations but obviously increased expression levels of BRG1 in 38 tumor samples (196). In addition, relatively high BRG1 expression is associated with the advanced stage and lymph node metastasis of gastric carcinoma (197), suggesting an oncogenic role for BRG1 in the gastric cancer setting. In addition, *BRG1* is reported as a pro-oncogenic element in neuroblastoma, where *BRG1* regulates multiple cellular processes, including cell growth, cell proliferation, cell cycle, and cell survival through oncogenic pathways such as BCL2 and PI3K/AKT (194), and BRG1 promotes breast cancer cell proliferation through multiple mechanisms, including fatty acid and lipid synthesis pathways, and ATP-binding cassette (ABC) transporter expression (192, 193). Hence, more studies are needed to provide deeper molecular basis for BRG1 functions and with the rationale for targeting the BRG1 oncoprotein as an effective therapeutic approach to treat cancers.

### SMARCB1

Loss of *SMARCB1* (*INI1*, *BAF47*) is usually present in childhood malignant rhabdoid tumors (MRT), a rare but lethal pediatric sarcomas characterized by a 22q11 chromosome rearrangement (198). Results from mouse models reveal that 10%–30% of heterozygous mice developed cancer, mainly soft tissue sarcomas and neurological diseases, indicating that *SMARCB1* is a tumor-suppressor gene (199–201). Mechanistically, *SMARCB1* deficiency causes most of the SWI/SNF complexes to be disassembled at the promoters and typical enhancers but has little effect on the super-enhancer-associated complexes (182).

Interactions between SMARCB1 and a large fraction of signaling pathways have been clarified, including the Wnt pathway, p16-RB pathway, and Sonic hedgehog (Shh) pathway (183). In *SMARCB1*-loss MRT cells, inhibition of EZH2 acts as a substitute for SMARCB1, which downregulates the expression of Shh pathway-associated genes, *EZH2* and *MYC*, and upregulates the expression of cell cycle inhibitors, neural differentiation genes, and tumor suppressors (157).

### PBRM1

*PBRM1* (also known as *BAF180*) mutation is observed in approximately 40% of renal cell carcinomas (RCC) and also in a small number of breast cancers (12, 184). In a large number of human tumors, expression of PBRM1 is negatively correlated with the expression of T-cell cytotoxicity genes and murine melanomas lacking *Pbrm1* are more likely to be infiltrated by cytotoxic T cells (185). Thus, the inactivation mutation of *PBRM1* may be more sensitive to immunotherapy, where cytotoxic T cells act as the main effector.

### Bromodomain-Containing 7

Bromodomain-containing 7 (BRD7) subunit is present uniquely in the PBAF complex, which usually serves as a tumor-suppressor gene and is downregulated in multiple cancers, such as breast cancer, HCC, ovarian cancer, and colorectal carcinoma (186, 202, 203). Previous studies have demonstrated that BRD7 may inhibit tumorigenicity *via* multiple mechanisms, including Ras/MEK/ERK, β-catenin, p53, and BRCA1 pathways (204). For instance, a study by Chen et al. identifies that *BRD7* inhibits cellular growth, motility, and cell cycling in HCC cells by positive regulation of p53 pathway activity (204). A variety of targets, such as estrogen receptor α (ERα), are regulated by BRD7 and BRCA1 coordinately in breast cancer, and BRD7 regulates ERα transcription by recruiting BRCA1 and Oct-1 to the promoter of ESR1 (the gene encoding ERα) (205). In addition, a genome-scale CRISPR/Cas9 screening study demonstrates that melanoma cells are sensitized to killing by T cells under the inactivation of >100 genes including *Brd7* (185).

### Summary

Given that the discovery of widespread SWI/SNF gene mutations in cancer is only a decade old, our understanding of the mechanisms and any corresponding therapeutic implications toward mutations in every subunit remains in its infancy. Although it is now clear that loss-of-function mutations affecting individual SWI/SNF subunits can confer specific dependencies on other genes or signaling pathways, whether any broad dependencies extend across all SWI/SNF cancers remains a key question, which merits further investigation. With repeated discoveries that mutations in SWI/SNF subunits often increase dependencies on other components of residual SWI/SNF complexes, growing interest surrounds the possibility to therapeutically target the SWI/SNF complex itself. The discovery of vulnerabilities, including both genetic and pharmacological, has been accelerated by the advances in high-throughput screening assays, which have been proved to be powerful for the identification of synthetic lethality and other similar relationships. Nevertheless, the promising results using cell line models need to and should be further verified by clinical trials.

## Conclusions

Under different circumstances, *ARID1A* plays a specific role in tumor initiation or tumor suppression. In this review, we discuss in detail the underlying mechanisms of ARID1A in tumor suppression, although *ARID1A* is also important in other biological processes related to tumors, including regulation of cell cycle, P53 target genes, and participation in DNA damage repair. By far, a variety of effective targets based on the synthetic lethality with *ARID1A* mutations have been discovered, and some drug candidates are in clinical development. Notwithstanding findings of ARID1A as a key tumor suppressor and potential therapeutic target, important gaps in knowledge remain concerning *ARID1A* mutations and deficiency in tumors. In summary, this review provides a more detailed understanding of the function of *ARID1A* in cancers, in order to assist and establish mouse models suitable for preclinical research to evaluate treatment efficiency and to further explore the therapeutic intervention methods for tumors with *ARID1A* mutations.

## Author Contributions

SX wrote the original manuscript. CT designed the structure of the manuscript and edited the manuscript. All authors contributed to the article and approved the submitted version.

## Conflict of Interest

The authors declare that the research was conducted in the absence of any commercial or financial relationships that could be construed as a potential conflict of interest.

## Publisher’s Note

All claims expressed in this article are solely those of the authors and do not necessarily represent those of their affiliated organizations, or those of the publisher, the editors and the reviewers. Any product that may be evaluated in this article, or claim that may be made by its manufacturer, is not guaranteed or endorsed by the publisher.
